# Chemoradiation of glioblastoma cells alters expression of activation and immune checkpoint molecules on type 1 and 2 dendritic cells and impacts on subsequent T cell proliferation

**DOI:** 10.1016/j.ctro.2025.101102

**Published:** 2026-01-09

**Authors:** Celina Schuster, Benjamin Frey, Rainer Fietkau, Stefanie Corradini, Udo S. Gaipl, Anja Derer

**Affiliations:** a Translational Radiobiology, Department of Radiation Oncology, Universitätsklinikum Erlangen, Friedrich-Alexander-Universität Erlangen-Nürnberg (FAU), Erlangen, Germany; b Department of Radiation Oncology, Universitätsklinikum Erlangen, Friedrich-Alexander-Universität Erlangen-Nürnberg (FAU), Erlangen, Germany; c Comprehensive Cancer Center Erlangen-EMN, Erlangen, Germany

**Keywords:** Glioblastoma, Chemoradiation, Dendritic cells, T cells, Immune checkpoint molecules, Chemokines, Cytokines

## Abstract

**Background and purpose:**

Glioblastoma is the most aggressive malignant brain tumor with an overall poor prognosis despite advanced chemoradiation approaches. Immunotherapeutic strategies have not been beneficial to date. Even being present only at low level in the brain, dendritic cells (DCs) have the potential to promote anti-tumor immune responses. In this study we aimed to gain insight into the interactions between glioblastoma tumor cells, DCs and T cells to understand the molecular and cellular mechanisms driving immune alterations in glioblastoma during standard treatments for optimizing immune therapies in the future.

**Material and methods:**

Impact of the conventional chemoradiation (RCT) treatment scheme of glioblastoma cells on conventional DCs type 1 and 2 (cDC) was tested in a syngeneic murine cell culture setting. GL261-luc2 glioblastoma cells were treated and subsequently co-cultured with cDC1-like and cDC2-like cells. Their immunological status was determined as follows: Expression of activation and immune checkpoint markers was quantified via flow cytometry. Cytokine and chemokine secretion was measured by bead-based immunoassays. Mixed lymphocyte reactions with either CD4+ or CD8+ T cells were performed to determine the potential of cDCs in stimulating T cell proliferation.

**Results:**

The cellular contact of cDC1-like cells with RCT-treated glioblastoma cells shifted their immune phenotype to a more activated one, whereas the activation of cDC2-like cells was limited. Furthermore, the extracellular profile of inflammatory and immunoregulatory cytokines and chemokines was highly dependent on the tumor cell treatment scheme in co-culture with cDC1-like cells, with the most pronounced effect after RCT. These modifications in the activation status of cDC1- and cDC2-like cells after tumor cell contact subsequently resulted in significantly enhanced CD8^+^ and CD4^+^ T cell proliferation.

**Conclusion:**

Current glioblastoma cell treatment impacts on the subsequent activation of cDCs and T cells and should serve as basis for improving immunotherapeutic strategies of brain tumors.

## Highlights

• Chemoradiation-treated glioblastoma cells increase activation and immune checkpoint molecules on cDC1-like cells.
• cDC2-like cells exhibit a comparatively minor response.
• The extracellular milieu's composition is dependent on the treatment regimen.
• T-cell proliferation increases due to modifications in dendritic cells after chemoradiation of glioblastoma cells.

## Introduction

Glioblastoma, categorized as WHO grade IV glioma, is the most common and aggressive primary brain tumor [1], [2]. It can be further subdivided into classical, mesenchymal, neural and proneural subtypes, based on unique molecular signatures [3], [4], although they share the wildtype isocitrate dehydrogenase 1/2 (IDH) status. Despite therapeutic approaches, the prognosis remains poor with a median survival of 15 months and a 5-year survival rate of less than 5% [2], [5], [6]. Due to its infiltrative growth, complete resection of glioblastoma is extremely difficult and consequently most tumors recur [7]. Therefore, the current standard of care consists of maximal safe surgical resection followed by fractionated radiotherapy (60 Gy, 1.8gy to 2 Gy fractions over a six-week time frame) and concurrent chemotherapy with temozolomide (TMZ) [7], [8], [9], which provides a better clinical outcome than radiotherapy alone [1]. The primary goal of ionizing radiation is to destroy cancer cells by causing DNA damage, preventing further cell proliferation, and inducing cell death [10]. TMZ achieves similar effects by alkylating DNA and causing nucleotide mismatches that lead to cell cycle arrest and cell death [11].

In addition to genetic and mutational impact, the tumor microenvironment contributes significantly to its heterogenous nature. For an extended period, the central nervous system (CNS), particularly the brain, has been described as “immune-privileged” [5], [12] and glioblastoma as a “cold tumor” due to its minimal lymphocytic infiltration and immunosuppressive phenotype, which poses challenges for immunotherapeutic strategies [4]. However, despite the protective blood–brain barrier, the CNS possesses active immune surveillance with different immune cell populations involved [13], chemokine and cytokine secretion [10] and immune checkpoint expression, e.g. programmed cell death protein 1 (PD-1) and cytotoxic T-lymphocyte-associated Protein 4 (CTLA-4) [14]. Tumor-associated macrophages (TAMs) constitute the predominant immune cell population within glioblastoma tumor tissue, comprising 30–40% of the total cell composition. Furthermore, regulatory T cells (Tregs) have been shown to suppress antitumor immunity [15]. However, two other immune cell types within the tumor microenvironment (TME) have been identified as playing a pivotal role in anti-tumor immune responses: Antigen-presenting dendritic cells (DCs) priming T cells (TCs) [16], [17]. The net effect on the tumor, whether pro- or anti-tumorigenic, is contingent on the specific subtypes of DCs and TCs involved. DCs are derived from hematopoietic progenitor cells within the bone marrow, undergoing various stages of differentiation through the stimulation of cytokines [13]. Finally, committed DC precursors give rise to conventional DCs (cDCs) and plasmacytoid DCs (pDCs). While pDCs are recognized as the primary producers of type I interferons, cDCs fulfill a substantial function in antigen presentation [18]. cDCs can further be sub-divided into cDC1- and cDC2-like cells. The classification of these cells is primarily determined by the expression of surface proteins. Both cell types express common myeloid molecules, such as MHCII and CD11c. However, cDC1-like DCs are further characterized by the expression of CD103, XCR1, and BTLA, and are therefore referred to as CD103^+^ cDCs. Conversely, -like DCs do not express these markers and are therefore designated as CD103^−^ cDCs [19]. While cDC1-like cells primarily engage in cross-presentation of antigens on MHCI, thereby activating CD8^+^ cytotoxic T cells, cDC2-like cells promote an augmentation in MHCII antigen presentation and CD4^+^ T helper cell activation [16], [20], [21]. Consequently, cDC1-like cells are the primary cells responsible for anti-tumor immunity, while cDC2-like cells serve a more regulatory function. cDC1-like cells can also be licensed by CD4^+^ T cells to prime CD8^+^ T cells, which are capable of killing tumor cells [22].

With respect to the immunological interactions in glioblastoma and its microenvironment, several immunotherapeutic approaches have been established, including peptide vaccines, DC vaccines, chimeric antigen receptor (CAR) T cell therapy, oncolytic virotherapy, and checkpoint inhibitors [5], [23]. Unfortunately, in contrast to many solid tumor types, glioblastoma exhibits a high degree of resistance to these immunotherapeutic strategies to date [12].

The objective of this study was therefore to achieve a more profound comprehension of the impact of current therapeutic modalities on the interaction between glioblastoma cells and DCs, and the potential subsequent effect on T cells. To this end, we conducted a comprehensive analysis of the immunogenic phenotype of cDC1- and cDC2-like cells. Our analysis encompassed the expression of activation and checkpoint markers, as well as the secretion of chemokines and cytokines following contact with glioblastoma cells that had been subjected to conventional treatments. To further investigate the role of cDC activation in modulating T cell activation, we conducted mixed lymphocyte reactions. Additionally, to ascertain the potential role of PD-1 blockade in these interactions, it was incorporated into all functional assays.

## Material and methods

### Animals

For the primary cell isolation, wild type C57BL/6 (for DC generation) and BALB/c (for T cell isolation) mice were procured from JANVIER LABS and maintained under sterile conditions at the preclinical experimental animal center (PETZ) of the University of Erlangen-Nuremberg. All experiments were conducted with male mice of three to four months of age. All experiments were approved by the local ethics committee.

### Cultivation and treatment of murine GL261-luc2 glioblastoma cells

The murine GL261-luc2 glioblastoma cell line (Caliper LifeSciences) was utilized in all experiments. The cells were cultivated in Dulbecco's Modified Eagle Medium (DMEM; Gibco™) with 10% heat-inactivated fetal calf serum (FCS; Sigma-Aldrich) and 0.5% geneticin (Gibco™) in a humidified chamber at 37°C and 5% CO_2_. Once the cells had reached at least 80% confluence, they were passaged for continuous cultivation or harvested, counted, and reseeded for further treatment. The cells were transferred into 75 cm^2^ flasks containing 15 ml of fresh culture medium, with each flask containing 3 × 10^5^ cells. The cells were then subjected to either chemotherapy (CT), radiotherapy (RT), or combined radio-chemotherapy (RCT), according to the treatment scheme outlined in Suppl. Fig. S1A. One flask was maintained as an untreated control (mock). The hypo-fractionated RT was administered in two doses of 5 Gy (Gy) via X ray generator (Seifert ISOVOLT) on days two and five. The administration of temozolomide (TMZ; Sigma-Aldrich) was carried out over a period of five consecutive days, with a dosage of 20 μM. On day six, cell culture media and GL261-luc2 cells were harvested in an independent manner, contingent upon distinct treatment conditions.

### Generation of differentiated murine cDC1- and cDC2-like cells

To initiate cDC differentiation, bone marrow was isolated from femora and tibia of C57BL/6 mice by flushing the bones with a 27G cannula. A single cell suspension was prepared, cells were subjected to centrifugation (350 g; 5 min), and resuspended in fresh DC medium. DC medium comprised of RPMI 1640 (Sigma-Aldrich) supplemented with 10% heat inactivated FCS (Sigma-Aldrich), 1% PenStrep, 0.1% ß-mercaptoethanol and 1 mM sodium pyruvate (all Gibco™). Cells were counted using a Neubauer counting chamber.

A modified protocol of Mayer et al. [24] was utilized to differentiate cDC1-like cells (CD103^+^). After counting, bone marrow-derived cells were seeded and incubated at a density of 1.5 × 10^6^ cells/ml in 10 ml DC medium with freshly added 1.5 ng/ml rmGM-CSF (Miltenyi Biotec) and 100 ng/ml rhFLT3L (Miltenyi Biotec). On day five, 5 ml of fresh DC medium (without cytokines) was added. On day nine, non-adherent cells were harvested, counted and reseeded at 0.3 × 10^6^ cells/ml in DC medium containing 1.5 ng/ml rmGM-CSF and 100 ng/ml rhFLT3L. On day twelve, cells were harvested, washed, counted and resuspended at 1 × 10^7^ cells/ml for co-culture with conditioned medium and GL261-luc2 cells.

A similar procedure was performed for cDC2-like (CD103^-^) cell differentiation, following a protocol developed by Lutz et al [25]. Cells were seeded at 2 × 10^6^ cells/ml in 10 ml DC medium containing 4 ng/ml rmGM-CSF. On day three of incubation, 5 ml of fresh DC medium containing 4 ng/ml rmGM-CSF was added. One day later, the cells were harvested, centrifuged, resuspended in 10 ml fresh medium containing 4 ng/ml rmGM-CSF and returned to the original dishes. Finally, on day seven, non-adherent cells were collected, centrifuged and resuspended at 1 × 10^7^ cells/ml for further usage (Suppl. Fig. S1B).

### Conditioned medium co-culture assay of GL261-luc2 and cDC1- or cDC2-like cells

For the conditioned medium co-culture assay, the cells were subjected to an incubation with either 10 μg/ml anti-IgG2a isotype or anti-PD-1 antibody (BIOZOL). Then, 1 ml of the conditioned medium was transferred into the respected wells of a 24-well-suspension plate (Greiner). Differentiated cDC-like and GL261-luc2 cells were added in a 2:1 ratio. As assay internal controls, cDC samples remained with antibody solely (negative control) or 1 μg/ml lipopolysaccharide (LPS, Sigma-Aldrich) stimulation (positive control). Cell analyses were conducted following a 24-hour incubation via flow cytometry.

### CD4^+^ and CD8^+^ T cell isolation from murine spleen and CFSE staining

The creation of an allogeneic system, involved the isolation of T cells from the spleens of BALB/c mice. Initially, a single cell suspension was obtained by meticulously dissecting the spleen and subsequently passing the tissue through a 70 μm cell strainer into a tube containing medium. Subsequently, the splenic cells were subjected to centrifugation and erythrocyte lysis. Thereafter, the cell number was determined, cells were magnetically labeled and separation of T cells was performed according to the manufacturer's instructions for CD^8+^ or CD4^+^ T cell isolation (Miltenyi Biotec). After purification, isolated T cells underwent staining with 1 μg/ml Carboxyfluorescein succinimidyl ester (CFSE, eBioscienceTM) for a duration of 30 min in a humidified chamber maintained at 37°C and 5% CO_2_, washed via centrifugation and resuspended in T cell medium. The T cell medium comprised RPMI 1640 (Sigma-Aldrich), with supplementation of 10% heat-inactivated FCS (Sigma-Aldrich) and 1% PenStrep (Gibco™).

### Mixed lymphocyte reaction (MLR)

For the mixed lymphocyte reaction (MLR), GL261-luc2 glioblastoma cells, cDC-like cells and T cells, were co-cultured. According to immunological activation cascades, either cDC1-like cells were combined with CD8^+^ T cells or cDC2-like cells were joint with CD4^+^ T cells. Therefore, 50 μl of pre-differentiated cDCs were seeded into a 96-well U-bottom plate and (treated) GL261-luc2 cells were added in a 2:1 ratio. After 24 h of incubation, co-cultured cells were irradiated with a single high dose of 25 Gy to induce cell cycle arrest and prevent further proliferation. Then, 100 µl of CFSE-stained T cells were added per well. Some wells were left as negative controls (all cell types only) or as positive controls (cDCs and T cells stimulated with LPS or Concanavalin A (ConA, Sigma-Aldrich). After two and four days of incubation, the MLR was mixed via careful pipetting to allow further cell proliferation. On day six, cells and supernatants were collected; supernatants were stored at −80°C for later purposes and cells were used for flow cytometric analyses. The CFSE-staining enabled the detection of different T cell daughter generations (F); the gating strategy is shown in Fig. S5.

### Flow cytometric analyses of cell surface markers and T cell proliferation

Cells were left unstained in FACS buffer (PBS Gibco™; 10% FCS), combined with a vitality dye (Zombie NIR^TM^ (BioLegend) or stained with different antibody panels. To analyze the conditioned medium co-culture assay, three antibody panels were used. To evaluate the cytokine-based differentiation of cDC1- and cDC2-like cells, CD11c (BD), MHCII (eBioscience), CD103 (BioLegend), XCR1 (BioLegend) and BTLA (eBioscience) were added. The gating strategy is shown in Suppl. Fig. S2 the quantification in Suppl. Fig. S3. To determine the activation of cDCs the following antibody panel was used: MHCII (eBioscience), CD11c (BD), CD40 (eBioscience), CD80 (BD), CD86 (BD). The third panel containing PD-1 (BD), ICOS-L (BioLegend), HVEM (BioLegend), Ox40L (eBioscience) and TIM-3 (eBioscience) antibodies was used to examine immune checkpoint marker expression of both DC subtypes. The MLR assays were stained with CD8a (BD), CD4 (BD), CD25 (BD) and CD3e (BioLegend) antibodies. Cell staining was performed for 30 min at +4°C in the dark. After washing and resuspending cells in FACS buffer, samples were analyzed using a CytoFLEX S flow cytometer (Beckman Coulter Life Sciences). Flow cytometric analysis was performed using Kaluza analysis software (Beckman Coulter Life Sciences).

### Multiplex array of secreted cytokines, chemokines, and HSP70 ELISA

Collected supernatants from conditioned medium co-culture assays were analyzed for cytokine and chemokine secretion. For these bead-based multiplex immunoassays, BioLegend's predefined LEGENDplex™ panels were used. Both the “Mouse Inflammation Panel” (Cat. No. 740446) and the “Mouse Proinflammatory Chemokine Panel” (Cat. No. 740451) were capable of detecting up to 13 analytes by flow cytometry. The arrays were used according to manufacturer’s instructions. For comparison analyses of cytokine secretion, the level of expression was normalized for co-culture of (i) cDCs only compared to cDCs with mock-treated GL261-luc2 and for (ii) cDCs only with mock-treated versus cDCs only with RCT-treated GL261-luc2 cells., values were normalized to the corresponding control condition of the same experiment, which was set to 1 (arbitrary units), and the values of the comparison groups were expressed relative to this reference. In addition, supernatants were analyzed for the presence of HSP70, a damage-associated molecular pattern (DAMP) protein, according to the manufacturer's instructions (abcam).

### Statistical analysis

Flow cytometric data were analyzed using Kaluza Analysis software. GraphPad Prism software (version 9.5.1) was used for statistical calculations and graphical presentation. Statistical analysis between groups was performed via 2-way ANOVA with Bonferronís correction test for multiple comparisons (0.12 (ns), 0.033 (*), 0.002 (**), <0.001(***), unless stated otherwise.

## Results

### cDC1-like cells significantly increase their surface expression of activation markers and immune checkpoint molecules upon contact with chemoradiation-treated glioblastoma cells

**Fig. 1:**
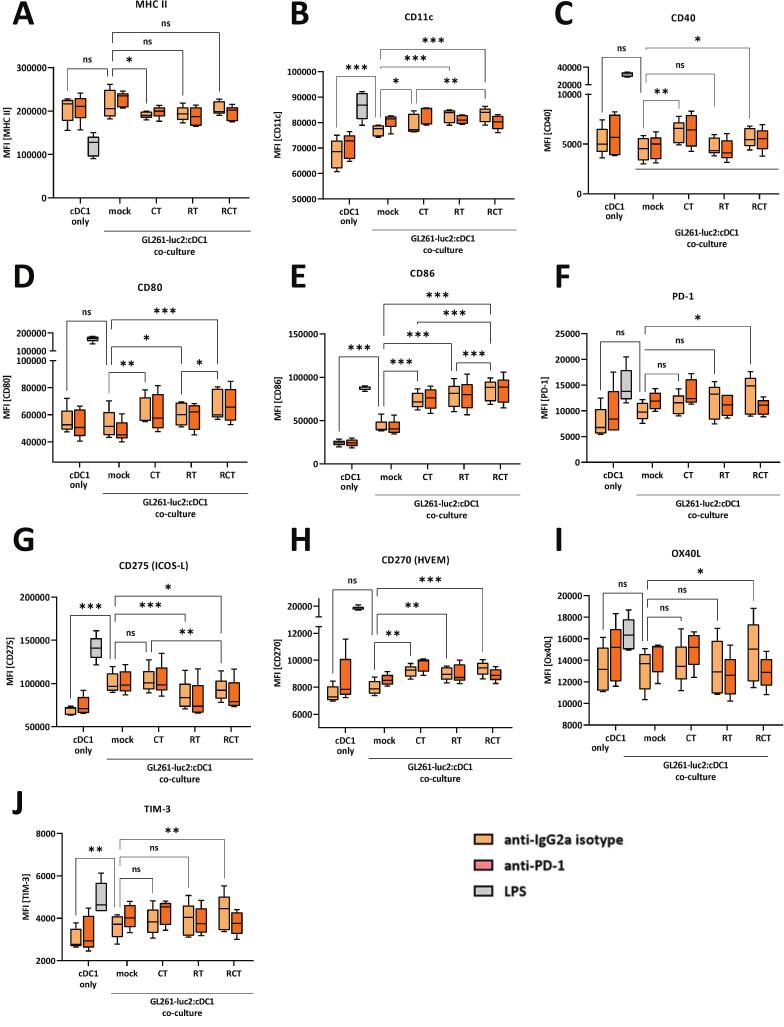
Pre-differentiated cDC1-like cells enhance their expression of activation markers and immune checkpoint molecules after contact to chemoradiation-treated glioblastoma cells. Flow cytometric expression and quantification analyses of surface proteins (A) MHCII, (B) CD11c, (C) CD40, (D) CD80, (E) CD86, (F) PD-1, (G) ICOS-L, (H) HVEM, (I) OX40L and (J) TIM-3 of either cDC1-like cells only or in a co-culture assay with pre-treated GL261-luc2 cells. Mono-cultured cDC1-like cells severed as baseline and were cultured with either anti-IgG2a (isotype) or anti-PD-1 antibody or stimulated via LPS. Treatment of tumor cells included mock (untreated), CT (TMZ), RT (2 × 5 Gy) or RCT (TMZ plus 2 × 5 Gy). Summary data from five independent experiments (n = 5) were plotted as Box with Min to Max; statistical analyses between the groups were done via 2way ANOVA with Bonferronís correction for multiple comparisons (0.12 (ns), 0.033 (*), 0.002 (**), <0.001(***). *Abbrevations:* cDC, conventional dendritic cell; CD, cluster of differentiation; HVEM, Herpesvirus entry mediator; ICOS-L, inducible costimulatory-ligand; LPS, lipopolysaccharide; MHC, major histocompatibility complex; OX40L, OX40 ligand; PD-1, programmed cell death protein; TIM, transmembrane immunoglobulin and munin domain.

Following antigen uptake, DCs initiate a maturation process characterized by increased expression of costimulatory molecules such as CD40, CD80, and CD86 [26], hereafter referred to as activation markers. In addition, DCs express immune checkpoint molecules such as PD-1, ICOS-L, HVEM, Ox40L and TIM-3, which are able to control anti-tumor responses [27], [28]. We aimed to decipher the effect of cell–cell contact of standard chemoradiation-treated glioblastoma cells on cDC1-like cells regarding their activation and immune status. Therefore, we treated GL261-luc2 glioblastoma cells with CT (TMZ) or RT alone or with the combined RCT (Fig.S1A). Untreated cells were used as mock controls, and LPS stimulation was used as an assay-internal positive control. We then co-cultured these treated tumor cells with pre-differentiated cDC1-like cells and examined the cell surface expression of DC activation markers (Fig. 1, A-E) and immune checkpoint molecules by flow cytometric analysis (Fig. 1, F-J). Contact of cDC1-like cells with pure untreated tumor cells only slightly increased the expression of CD11c and CD86 (Fig. 1, B, E). However, mono- and combination-treatment of glioblastoma cells strongly affected the expression level of cDC1-like cells. CT reduced MHC II (Fig. 1A), but increased the expression of CD11c, CD40, CD80 and CD86 (Fig. 1B–E). RT alone induced CD11c, CD80 and CD86 (Fig. 1B, D-E). Combined chemoradiation had the greatest effect, inducing CD11c, CD40, CD80 and CD86 (Fig. 1B-E), with CD86 being the most prominent receptor to be altered. A similar pattern was observed for the expression of immune checkpoint markers: Co-culture of mock tumor cells with cDC1-like cells slightly induced ICOS-L and TIM-3 expression (Fig. 1G, J). Single CT or RT had no effect on PD-1, Ox40L and TIM-3 expression (Fig. 1F, I-J). RT reduced ICOS-L and induced HVEM expression (Fig. 1G-H) compared to the sham co-culture condition. However, the most important finding was the cumulative effect of combined chemoradiation of glioblastoma cells, where all immune checkpoint molecules tested were significantly altered in their surface expression on cDC1-like cells when compared to untreated tumor cells and cDC1-like cell co-culture: ICOS-L was downregulated, whereas PD-1, HVEM, Ox40L and TIM-3 were upregulated (Fig. 1F-J). Notably, the addition of an anti-PD-1 therapeutic antibody prior to co-culture had no effect on the expression profile of cDC1-like cells (Fig. 1A-J).

### Chemoradiation treatment of glioblastoma cells has limited effects on cDC2-like cells activation marker and immune checkpoint expression after tumor cell contact

**Fig. 2:**
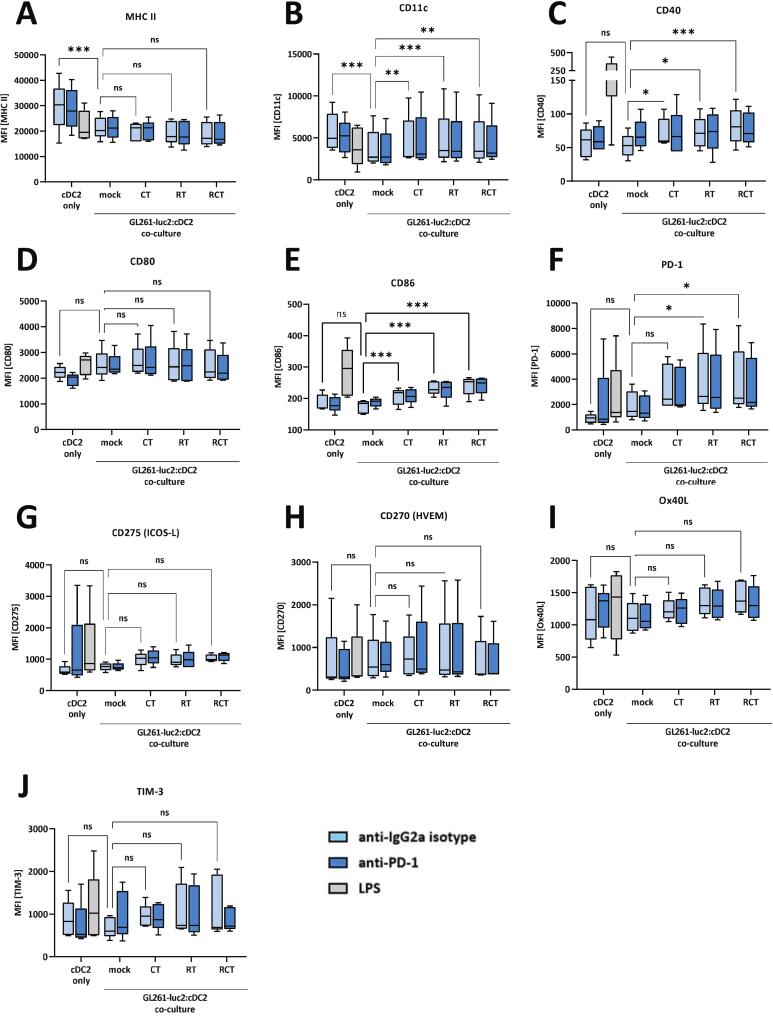
Chemoradiation treatment of glioblastoma cells has only limited effects on activation marker and immune checkpoint molecule expression of pre-differentiated cDC2-like cells after tumor cell contact. Flow cytometric expression and quantification analyses of (A) MHCII, (B) CD11c, (C) CD40, (D) CD80, (E) CD86, (F) PD-1, (G) ICOS-L, (H) HVEM, (I) OX40L and (J) TIM-3 of either cDC2-like cells only or in a co-culture assay with pre-treated GL261-luc2 cells. Mono-cultured cDC2-like cells severed as baseline and were cultured with either anti-IgG2a (isotype) or anti-PD-1 antibody or stimulated via LPS. Tumor cell treatment included mock (untreated), CT (TMZ), RT (2 × 5 Gy) or RCT (TMZ plus 2 × 5 Gy). Summary data from five independent experiments (n = 5) were plotted as Box with Min to Max; statistical analyses between the groups were done via 2way ANOVA with Bonferronís correction for multiple comparisons (0.12 (ns), 0.033 (*), 0.002 (**), <0.001(***). *Abbrevations:* cDC, conventional dendritic cell; CD, cluster of differentiation; HVEM, Herpesvirus entry mediator; ICOS-L, inducible costimulatory-ligand; ligand; MHC, major histocompatibility complex; OX40L, OX40 ligand; PD-1, programmed cell death protein; TIM, transmembrane immunoglobulin and munin domain.

In addition to cDC1-like cells, there are cDC2-like cells, which have critical and context-dependent functions in immunity. They can also recognize, process and present antigens, balance immune responses and contribute to antitumor immunity [29]. However, in an immunosuppressive tumor microenvironment, their phenotype can be altered and they tend to induce tolerance and promote immunosuppression [29]. Therefore, in a second approach, we analyzed the impact of glioblastoma cell treatment on the cell surface expression of activation markers and immune checkpoints of pre-differentiated cDC2-like cells (Fig. 2A-J). Interestingly, contact of cDC2-like cells with untreated tumor cells significantly reduced the expression of MHCII and CD11c (Fig. 2A,B). Furthermore, similar effects as for cDC1-like cells were observed in the regulation of activation marker expression on cDC2-like cells in cocultures with treated glioblastoma cells. In particular, there was a gradual upregulation of CD11c (Fig. 2B), CD40 and CD86 levels on cDC2-like cells, most significantly after chemoradiation (Fig. 2C,E), whereas MHC II and CD80 expression were not affected by treatment (Fig. 2A,D). Overall, although some changes were statistically significant, the effect of glioblastoma cell treatment does not seem to affect the activation of cDC2-like as much as cDC1-like cells. Moreover, none of the considered immune checkpoint molecule expression was significantly altered by cDC2-like cells to tumor cell co-culture (Fig. 2G-J), except for PD-1 (Fig. 2F). PD-1 expression could be significantly induced by RT alone or combined RCT. Similar to cDC1-like cells, blockade of PD-1 by therapeutic antibody did not affect cDC2-like cell surface expression at all (Fig. 2).

In conclusion, cellular contact with glioblastoma cells altered the immune phenotype of cDC1-like and cDC2-like cells. Importantly, the degree of modification was dependent on the cDC subtype and the type of glioblastoma cell treatment, with the strongest induction of surface markers following chemoradiation.

### The composition of inflammatory and immunoregulatory cytokines and chemokines is highly dependent on the treatment of glioblastoma cells

**Fig. 3:**
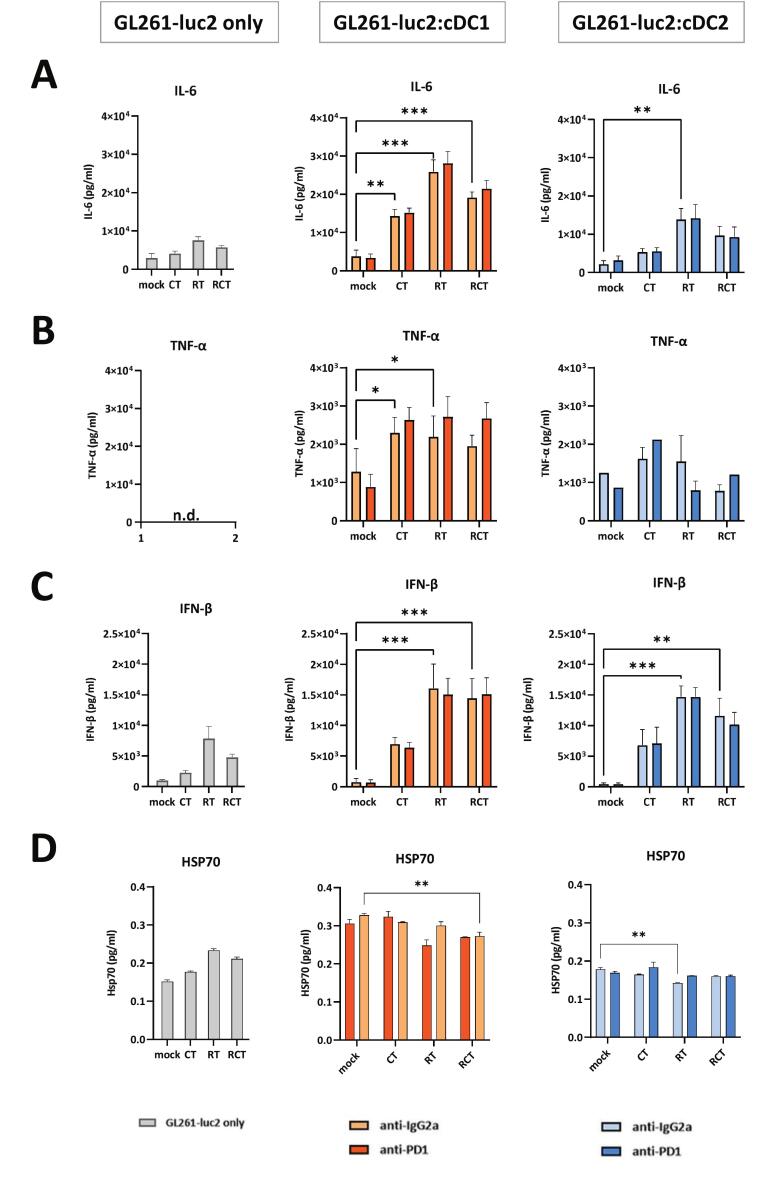
Secretion of pro-inflammatory is induced by contact of treated glioblastoma cells with cDC1-like and cDC2-like cells in a distinct manner and independent of PD-1. Cell culture supernatants of the different displayed conditions were quantified regarding the secretion of pro-inflammatory cytokines (A) IL-6, (B) TNF-α, (C) IFN-β, and (D) HSP70. The first column shows secretion of the respective cytokine within the GL261-luc2 mono-culture, the second and third columns show the results from co-culture assays of either cDC1-like or cDC2-like cells with treated GL261-luc2 cells. Treatment as indicated mock (untreated), CT (TMZ), RT (2 × 5 Gy) or RCT (TMZ plus 2 × 5 Gy). Summary data from four independent experiments (n = 4) were plotted as Mean ± SEM. Statistical analyses between the groups were done either via 2way ANOVA or mixed-effects analysis (due to missing, undetectable values) with Dunnett́s correction for multiple comparisons (0.12 (ns), 0.033 (*), 0.002 (**), <0.001(***). *Abbrevations:* cDC, conventional dendritic cell; IFN- β, interferon-β; IL, interleukin; LPS, lipopolysaccharide; TNF-α, tumor necrosis factor α.

**Fig. 4:**
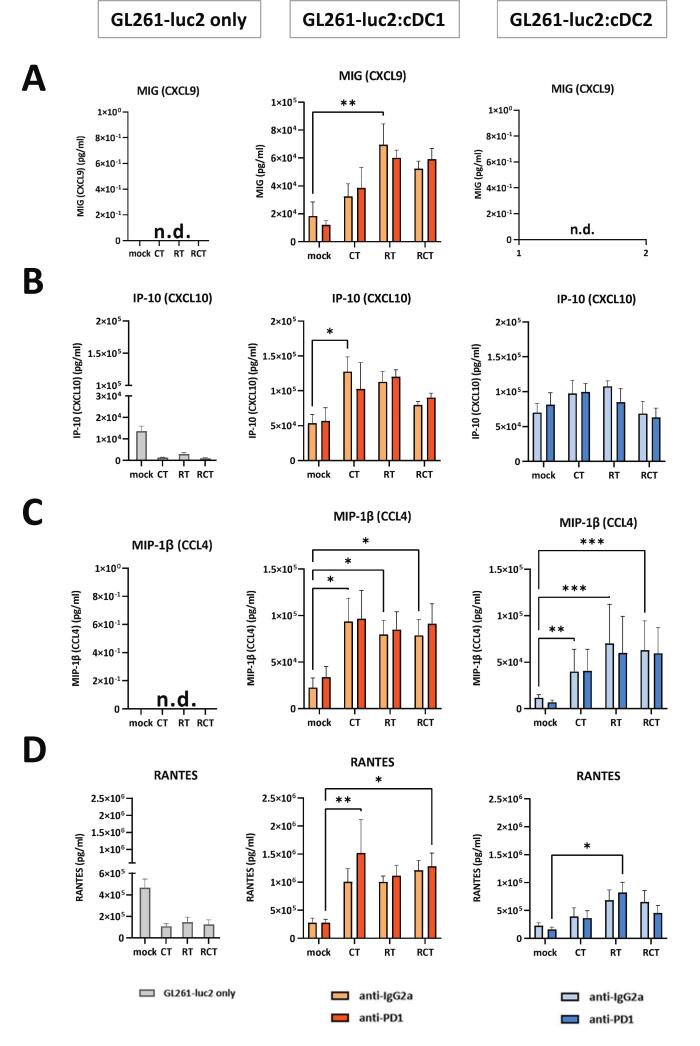
Secretion of immunoregulatory chemokines is induced by contact of treated glioblastoma cells with cDC1- and cDC2-like cells in a distinct manner and independent of PD-1. Cell culture supernatants of the different displayed conditions were quantified regarding the secretion of chemokines (A) CXCL9, (B) CXCL10, (C) CCL4 and (D) RANTES. The first column shows secretion of the respected cytokine within the GL261-luc2 mono-culture, the second and third column shows the results from co-culture assays of either cDC1-like or cDC2-like cells with treated GL261-luc2 cells. Treatment as indicated mock (untreated), CT (TMZ), RT (2 × 5 Gy) or RCT (TMZ plus 2 × 5 Gy). Summary data from four independent experiments (n = 4) were ploted as Mean ± SEM. Statistical analyses between the groups were done either via 2way ANOVA or mixed-effects analysis (due to missing, undetectable values) with Dunnett́s correction for multiple comparisons (0.12 (ns), 0.033 (*), 0.002 (**), <0.001(***). *Abbrevations:* cDC, conventional dendritic cell; CCL, C-C motif ligand; CXCL, C-X-C motif; LPS, lipopolysaccharide; RANTES, Regulated And Normal T cell Expressed and Secreted.

In the dynamic tumor microenvironment, cytokines and chemokines act as central key molecules of the immune system, capable of regulating and controlling immune responses. They are involved in various tumor-related processes such as proliferation, metastasis and angiogenesis [30]. As such, they can be considered as indicators of whether a pro-inflammatory or rather an immunosuppressive environment develops during tumorigenesis and anti-tumor therapy. To further evaluate the dependence of cDC stimulation on tumor cell treatment we analyzed the supernatants of mono- and in co-cultures (tumor cells and/or cDCs) for the secretion of selected cytokines and chemokines in a bead-based immunoassay (Fig. 3, Fig. 4). In the following only those targets are shown and discussed that revealed significant changes depending on the culture conditions.

Starting with the secretion of the prominent proinflammatory cytokines IL-6, TNF-α and IFN-β (Fig. 3), it is evident, that only IL-6 and IFN-β are expressed by GL261-luc2 cells, showing an upregulation especially after RT (Fig. 3A,C). None of these cytokines could be detected in unstimulated cDC1- and cDC2-like cultures, whereas LPS induced all of them, with higher detection rates observed in the cDC2-like cell monoculture. Interestingly, cytokine levels showed dynamic changes under co-culture conditions. IL-6 and IFN-β secretion increased in both cocultures after treatment, reaching a peak after RT (Fig. 3A,C). TNF-α secretion was significantly increased after treatment in the GL261-luc2 to cDC1-like cell coculture, whereas no changes were detected in the cDC2-like cell coculture (Fig. 3B). A comparison of the two cDC subtypes revealed similar expression patterns, with higher levels of IL-6 and TNF-α protein within the cDC1-like cell conditions.

As a class of signaling molecules, chemokines regulate the recruitment of immune cells and thereby initiate immune responses [31]. Examination of the secretion levels of immunoregulatory chemokines CXCL9, CXCL10, CCL4 and CCL5 (RANTES) (Fig. 4) revealed remarkable treatment-dependent changes. CXCL10 and RANTES were secreted by untreated GL261-luc2 cells, which was reduced after treatment with either CT or RT or the combination (Fig. 4B,D), whereas other chemokines could not be detected. Differentiated but unstimulated cDC1- and cDC2-like cells did not release any of the chemokines analyzed, but could be induced by LPS stimulation. However, just like cytokines, chemokines were secreted at higher levels after contact with differently treated glioblastoma cells. Notably, CXCL9 could not be recorded in the cDC2-like cell coculture, whereas it was found in the coculture of cDC1-like and untreated GL261-luc2 cells and got significantly increased after RT (Fig. 4A). In contrast to monocultured tumor cells, CXCL10 level were upregulated when mock tumor cells came into contact with cDCs. Further significant upregulation was found after treatment, in this case especially CT (Fig. 4B). CCL4 and RANTES were secreted at similarly elevated levels after contact of CT-, RT- and RCT-treated glioblastoma cells with cDC1-like, whereas in the cDC2-like cell coculture the effect of RT and RCT dominated. (Fig. 4C,D).

Furthermore, there is a strong link between damage-associated molecular patterns (DAMPs) and DC activation. DAMPs released from stressed, damaged, or dying cells are recognized by pattern recognition receptors (PRRs) on DCs and act as potent activators of DC maturation and immune function; therefore, we examined the supernatants of mono- and co-cultures for HSP70 secretion (Fig. 3D). As expected, treatment of glioblastoma cells induced the release of HSP70, mainly due to irradiation. In contrast, co-cultivation of treated tumor cells with cDC1-like cells lead to a slight decrease in free HSP70 compared to mock-treated tumor cells. The same effect but milder could be observed for cDC2-like cells. The higher HSP70 levels in DC cocultures compared with tumor cells alone were most likely due to higher levels in cDC-only cultures (Fig. S4).

Overall, monocultures of pure pre-differentiated cDC1- and cDC2-like cells showed very limited to no cytokine and chemokine levels, whereas tumor cells alone produced IL-6, IFN-β, CXCL10 and RANTES. Notably, this was modulated by treatment (increase for IL-6 and IFN-β, decrease for CXCL10 and RANTES). In a coculture condition, all levels got significantly increased in a treatment-dependent manner, with the effect being stronger in cDC1-like cells. Blockade of the PD-L1/PD-1 axis had no effect on the cytokine levels.

### Chemoradiation of GL261-luc2 glioblastoma cells and cell contact to cDC1- and cDC2-like cells significantly increases CD8^+^ and CD4^+^ T cell proliferation

**Fig. 5:**
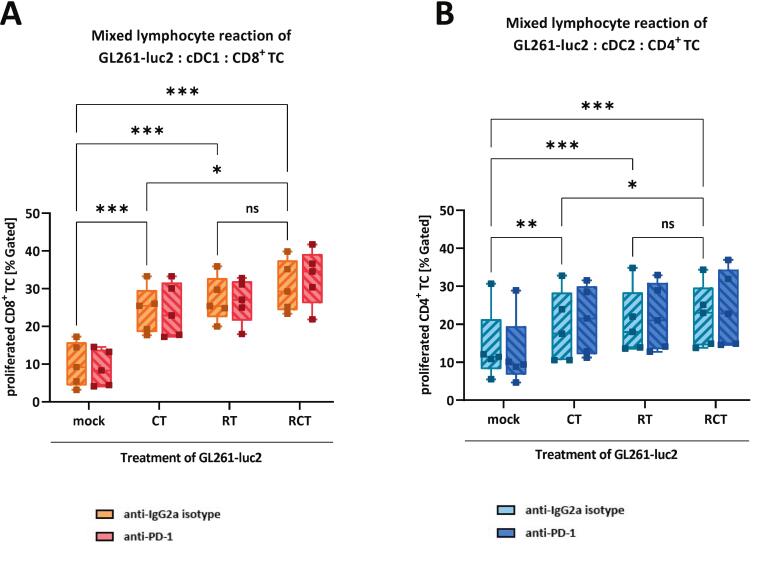
Treatment of glioblastoma cells enhances T cell activation by cDC1- and cDC2-like cells independent of the PD-1 axis. Mixed lymphocyte reaction assay of (A) cDC1-like cells, CD8+ T-cells and GL261-luc2 glioblastoma cells or (B) cDC2-like cells, CD4+ T-cells and GL261-luc2 glioblastoma cells. In addition, cultures were treated with either anti-IgG2a or anti-PD-1 antibody. Pre-treatment of GL261-luc2 as indicated via mock (untreated), CT (TMZ), RT (2 × 5 Gy) or RCT (TMZ plus 2 × 5 Gy). Summary data from five independent experiments (n = 5) were plotted as Box and Whiskers (Min to Max, with all data points showing). Statistical analyses between the groups were done via 2way ANOVA Bonferronís correction for multiple comparisons (0.12 (ns), 0.033 (*), 0.002 (**), <0.001(***). *Abbrevation:* cDC, conventional dendritic cell.

Having verified the phenotypic changes of cDC1- and cDC2-like cells after cell-to-cell contact with RCT-treated glioblastoma cells, we aimed to further evaluate whether this actually has subsequent consequences for functional T-cell activation. Therefore, we performed MLR assays with triple-co-culture of either GL261-luc2, cDC1-like and CD8^+^ T cells (Fig. 5A) or GL261-luc2, cDC2-like and CD4^+^T cells (Fig. 5B). Both settings showed a significant increase in T cell proliferation when tumor cells were pretreated, with the strongest effect when RCT-treated tumor cells were used. Remarkably, the combination of RT and CT on tumor cells resulted in significantly higher T cell proliferation than CT alone, indicating RT was the main driver. The overall proliferation rate of CD8^+^ T cells activated by cDC1-like cells (Fig. 5A) was higher than that of CD4^+^ T cells stimulated by cDC2-like cells (Fig. 5B). Again, this outcome was not dependent on PD-L1/PD-1 interaction, as shown by the comparison of the anti-IgG2a isotype antibody with the anti-PD1 blocking antibody.

## Discussion

### Overview and main findings

In this study, we investigated the impact of the standard glioblastoma therapies- temozolomide and irradiation- on the stimulation of the cDC1- and cDC2-like cell subtypes and subsequent T cell activation, in dependence on GL261 glioma cell treatment, in a murine *in vitro*/ex vivo model. Our data show that contact between GL261 glioblastoma cells and predifferentiated cDC1-like cells triggers significant activation and maturation, with distinct cytokine and chemokine outputs, and promotes CD8^+^ T-cell proliferation. In contrast, cDC2-like cells exhibit lower activation, altered chemokine production, and reduced capacity to support T-cell responses.

### Subset-specific DC activation by chemo-irradiated glioblastoma cells

**Fig. 6:**
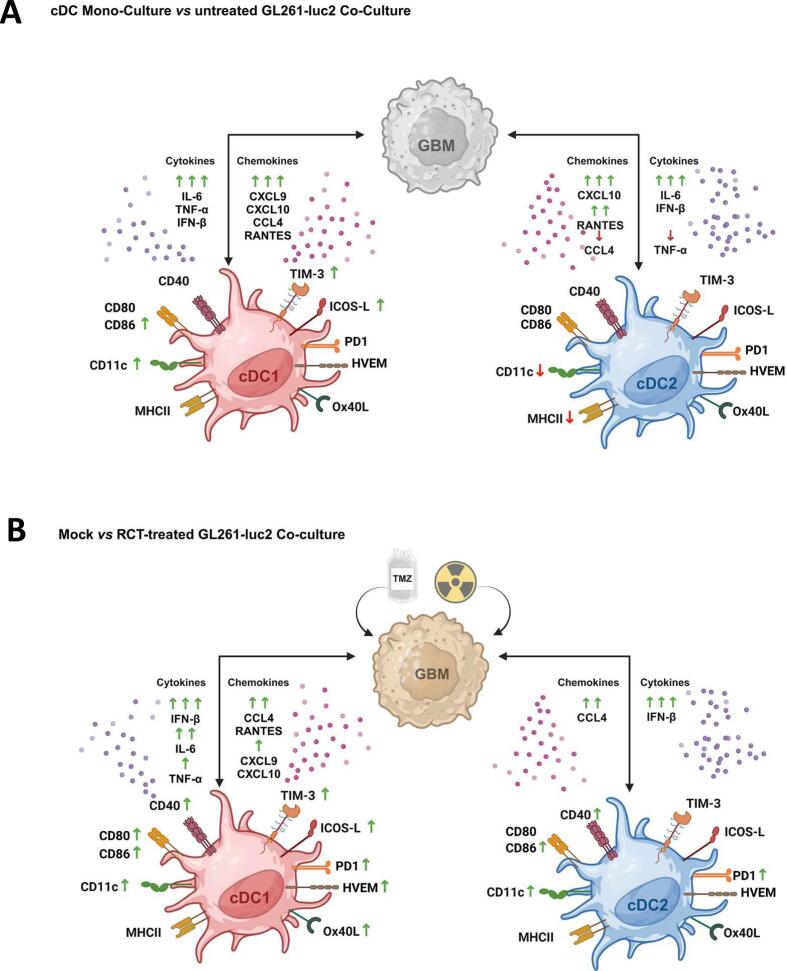
Schematic illustration of mechanistic effects on surface marker expression and cytokine release of co-cultivation of pre-differentiated cDCs and GL261-luc2 glioblastoma cells. (A) Untreated GL261-luc2 cells co-cultivated with cDC1-like increase the expression of surface proteins CD11c, CD86, TIM-3 and ICOS-L on cDC1-like cells and the secretion of IL-6, TNF-α, IFN-β, CXCL9, CXCL10, CCL4 and RANTES compared to pure cDC mono-culture. Cellular contact of tumor cells to cDC2-like cells decreases their expression of CD11c and MHCII; the secretion of CXCL10, RANTES, IL-6 and IFN-β is highly induced, whereas CCL4 and TNF-α are reduced. (B) Co-culture of mock vs. chemoradiation (RCT)-treated GL261-luc2 cells induces the expression of CD11c, CD80, CD86, CD40, TIM-3, ICOS-L, PD-1, HVEM and Ox40L of cDC1-like cells whereas on cDC2-like cells only the expressions of CD11c, CD86, CD40 and PD-1 are increased. RCT-treatment of tumor cells significantly enhances IFN-β, IL-6, TNF-α, CCL4, RANTES, CXCL9 and CXCL10 in the supernatant with cDC1-like cells, whereas the combination with cDC2-like cells only increases CCL4 and IFN-β. The level of expression was normalized for co-culture of (A) cDCs only compared to cDCs with mock-treated GL261-luc2 and for (B) cDCs only with mock-treated versus cDCs only with RCT-treated GL261-luc2 cells. *Legend:* Level of expression/secretion increase ↑ (1 – 3-fold), ↑↑ (3–6-fold), ↑↑↑ (>6-fold) or decrease ↓ (1 – 0,5-fold), ↓↓ (<0,5-fold). DC activation marker: CD11c, MHCII, CD40, CD80, CD86; Immune checkpoint protein: PD-1, ICOS-L, Ox40L, TIM-3, HVEM; Chemokines: CCL4, RANTES, CXCL9, CXCL10; Cytokines: IL-6, TNF-α, IFN-β. Created in BioRender. *Abbrevations:* cDC, conventional dendritic cell; CD, cluster of differentiation; CCL, C-C motif ligand; CXCL, C-X-C motif; HVEM, Herpesvirus entry mediator; ICOS-L, inducible costimulatory-ligand; ligand; IFN- β, interferon-β; IL, interleukin; MHC, major histocompatibility complex; OX40L, OX40 ligand; PD-1, programmed cell death protein; RANTES, Regulated And Normal T cell Expressed and Secreted; TIM, transmembrane immunoglobulin and munin domain; TNF-α, tumor necrosis factor α.

Cellular contact between GL261 glioblastoma cells and predifferentiated cDC1-like cells, particularly induced their expression of CD11c and CD86 (Fig. 6A). CD11c is a marker commonly associated with DCs and is involved in adhesion and migration. It can be described as an activation marker as it can be induced by immune signaling, cytokine responses, sensing, and maturation [32]. Factors such as type I and II interferons or pro-inflammatory cytokines such as TNF-α or IL-6 are known to induce CD11c expression [33], which aligns with the upregulated secretion of these proteins in the culture supernatant of cDC1-like cells (Fig. 6A). CDC2-like cells showed less CD11c expression after tumor cell contact, albeit IL-6 and IFN-β increased; however, TNF-α levels decreased, and the overall cytokine levels were lower compared to when cDC1-like cells were present. Another marker of DCs is CD86, a co-stimulatory receptor that is activated following pathogen recognition and the initiation of immune responses, including inflammation and DC maturation [34]. Its expression is induced by signals similar to those that trigger CD11c, which is why the simultaneous upregulation of CD11c and CD86 as indicators for cDC1-like cell activation and maturation after tumor cell contact is plausible. In contrast, the weaker modulation of these markers on cDC2-like cells suggests limited maturation. Overall, the expression levels of DC activation, co-stimulation, and immune checkpoint markers detected under co-culture conditions indicate a general activation and maturation of pre-differentiated cDC1-like cells upon contact with glioblastoma cells, consistent with the release of pro-inflammatory cytokines. Meanwhile, cDC2-like cells showed only a slight reduction in CD11c and MHC II expression, which more likely reflects maintenance of immune tolerance, suppression, or evasion strategies, possibly indicating either incomplete or defective maturation or a functional deficit in antigen presentation [35].

The RCT of glioblastoma appears to induce greater activation of dendritic cell subsets, particularly cDC1-like cells. This is shown by higher expression of co-stimulatory molecules, such as CD40, CD80, and CD86, in RCT-treated co-cultures compared to untreated controls, along with increased secretion of cytokines and chemokines, indicating a higher overall activation level and greater ability to stimulate T cells. Conversely, cDC2-like cells exhibit relatively limited changes in surface marker expression after co-culture with treated tumor cells, underscoring potential functional differences between these dendritic cell subtypes and supporting the idea that cDC1-like cells are the primary responders to chemoradiation-treated glioblastoma.

### Co-stimulatory and checkpoint molecules on DCs

Another co-stimulatory molecule reflecting DC activation and maturation is ICOS-L. It can be induced by TLR activation or by pro-inflammatory cytokines such as IL-4, IFN-γ, and TNF-α, and binds to its receptor, ICOS, on activated T cells to promote effective immune responses, particularly the differentiation and function of T follicular helper cells, which are crucial for humoral immunity [36]. In the context of TME, ICOS-L regulation of cDC1-like cells may contribute to tumor immune evasion by modulating immune responses. Its upregulation under coculture conditions was most likely a consequence of cDC1-like cell activation and further development. Concurrently, we found that TIM-3 expression was increased on cDC1-like cells. TIM-3, an immune checkpoint mainly found on T cells, but also expressed by DCs, is associated with immune regulation, tolerance, and immune exhaustion, and is often found upregulated as a counter mechanism for immune activation to balance and maintain immune homeostasis. TIM-3 expression on DCs, especially cDC1-like cells, can be induced by infection and inflammation, as well as by immunosuppressive mechanisms within a tumor [37]. Thus, the upregulation of TIM-3 after tumor cell contact could be due to general DC activation as a counter-regulatory response and/or to the immunosuppressive microenvironment generated by glioblastoma cells, and may indicate a shift of the cDC1-like subtype toward a more immunomodulatory phenotype that was not observed in cDC2-like cells.

Chemoradiation further increased the expression of activation markers and immune checkpoint proteins, particularly on cDC1-like cells. Co-cultivation of cDC1-like cells with chemoradiation-treated glioblastoma cells significantly enhanced external expression of CD80, CD86, and additional immune checkpoints such as PD-L1, PD-1, HVEM, OX40L, and TIM-3, and intensified cytokine and chemokine secretion, emphasizing that these cells adopt a highly activated yet checkpoint-rich phenotype under RCT conditions. The upregulation of immune checkpoints such as PD L1 on DCs can be a hint for activation but also for subsequent functional exhaustion, because of their natural role in negative feedback to prevent exaggerated immune responses. This may simultaneously restrain T-cell function, reflecting a complex balance between immune activation and suppression.

### Cytokine and chemokine networks

CCL4 (MIP-1β) and RANTES (CCL5) are potent chemokines that attract immune cells, such as CD8+ T cells and NK cells, to sites of infection and inflammation [38], [39]. In particular, CD8+ T cells can be guided by these chemokines, subsequently activated, and differentiated into functional cytotoxic T cells. In our system, CCL4 was not detected in tumor cells alone, whereas RANTES were secreted at relatively high levels (Fig. 4C,D). Both chemokines were significantly induced in the presence of cDC1-like cells in co-culture, while contact with cDC2-like cells did not alter RANTES secretion and even reduced CCL4, suggesting that poor activation of cDC2-like cells limits their contribution to chemokine-mediated effector cell recruitment. Like cDC1-like cells, cDC2-like cells can secrete CCL4 and RANTES to attract CD4^+^ T cells, as well as CD8+ T cells and NK cells; however, the reduced chemokine levels in cDC2-like culture conditions likely reflect their overall weaker activation state following tumor cell contact.

CXCL9 and CXCL10 play a pivotal role in inflammation and are potent Th1 as well as CTL and NK cell-attracting chemokines, so their presence is another indicator of immune activation [40]. Major inducers of CXCL10 are type I interferons [41], which may account for the high levels of CXCL10 in both cDC1-like and cDC2-like co-cultures. In tumor immunity, upregulation of these chemokines has been associated with better anti-tumor immune responses and improved patient outcomes [42], [43], and their production by cDC1-like cells, in particular, supports the recruitment and positioning of CD8+ T cells in the TME.

In general, treatment-induced changes in cytokine and chemokine secretion following DC–tumor cell interactions can shift the tumor microenvironment toward either an inflammatory or an immunosuppressive state, depending on the specific mediators involved. On the pro-inflammatory, anti-tumoral side, all relevant cytokines were already strongly induced after contact between cDC1-like cells and untreated glioblastoma cells, and this induction was further enhanced when tumor cells were treated, suggesting that RCT could markedly influence immune activation in vivo. Treatment-induced upregulation of CCR7 on dendritic cells promotes their migration to lymph nodes, where interaction with chemokines such as CCL19 facilitates efficient priming of CD4^+^ and CD8^+^ T cells, while elevated IL-12 further enhances T-cell activation and effector function [44], [45], [46]. At the same time, increased intratumoral expression of CXCL9, CXCL10, CCL4, and CCL5 after chemotherapy supports recruitment of dendritic cells and cytotoxic T cells into the tumor, with cDC1-like-derived CXCL9/10 and IL-12 reinforcing antitumor activity [47], [48].

In contrast, the same chemokine and cytokine networks can also foster immunosuppressive conditions, as their pleiotropic actions influence the composition and functional state of immune cells in the TME. Elevated IL-10, TGF-β, or CCL22 can promote regulatory T-cell accumulation and immune evasion [49], [50], and specific chemokine profiles may favor recruitment of additional suppressive cell populations, so that treatment-driven modulation of cytokines and chemokines represents a double-edged sword, in which balanced pro-inflammatory signals support T–cell–mediated tumor rejection. But an imbalanced or skewed profile contributes to chronic inflammation, immune suppression, and glioblastoma progression. Thus, a deeper understanding of individual mediators and their interplay is needed to target specific shifts toward anti-tumor immune responses.

### Mechanisms underlying differential DC activation

The mechanisms behind DC activation are complex and involve various signaling pathways, including cytokine networks, DAMP-mediated activation, and the cGAS-STING pathway. Specific cytokines are crucial for DC development and activation. FLT3L drives differentiation and expansion of conventional DCs, particularly cDC1, while IL-12 and type I interferons produced by activated DCs enhance their maturation and promote type 1–polarized T-cell responses [[51], [52], [53]], maturation, and their immunogenic functions, while Proinflammatory cytokines such as IL-6 and TNF-α contribute to DC maturation across subsets, including support for cDC2 activation in inflammatory contexts [[54], [55]]. In our experiments, we detected increased levels of IFN-β, IL-6, and TNF-α in the supernatants, which depended on tumor cell treatment and likely contributed to activating both cDC1- and cDC2-like cells. Notably, these cytokines were more prominently expressed in cultures of cDC1-like cells. Specifically, TNF-α is recognized for promoting the upregulation of costimulatory molecules such as CD80, CD86, and CD40, thereby boosting DC antigen presentation and T cell activation. These findings support our data and indicate cytokine-dependent differences in DC behavior activation.

The observed effects can also be linked to DAMP-triggered molecular and signaling pathways. DAMPs are internal molecules released by cells in response to stress or damage, such as during irradiation or other forms of cell death. These molecules act as danger signals and are essential mediators for activating the innate immune response in sterile inflammation, tissue repair, and immune surveillance. DCs recognize DAMPs through pattern recognition receptors (PRRs), including Toll-like receptors (TLRs), which initiate signaling cascades that lead to the transcription of pro-inflammatory cytokines and costimulatory molecules. Some DAMPs, like HSP70, are especially effective at inducing DC maturation, evidenced by increased levels of MHC class II, CD80, and CD86 on the cell surface, as well as increased secretion of cytokines such as IFN-γ and IL-12. In our experiments, we observed higher secretion of HSP70 from tumor cells following chemoradiation (Fig. 3D). Contact with these cells and conditioned medium from treated tumor cells significantly increased the expression of CD80 (in cDC1-like cells) and CD86 (in both cDC1- and cDC2-like cells) compared to untreated tumor cells (Fig. 1, Fig. 2, Fig. 6), suggesting a possible HSP70-dependent maturation process. The DAMPs released during chemoradiation not only activate DCs but also stimulate other immune cells, such as T cells, which would be in line with our findings [[56], [57]]. Nevertheless, several other DAMPs may be responsible for differential activation, underscoring the need for further investigation.

Another potential mechanism underlying differential DC activation is cytosolic DNA sensing via the cGAS-STING pathway [58]. The cGAS enzyme detects DNA from dying or damaged cells and produces cGAMP, which activates STING. This leads to the secretion of type I interferons and pro-inflammatory cytokines. cDC1-like cells are exceptionally responsive to cGAS-STING activation, producing robust IFN-I, which may contribute to their more pronounced activation. Further analyses via STING knockdown or cytokine neutralization are necessary to gain deeper mechanistic insights into the relative contributions of these pathways to the differential activation of cDC1 and cDC2 subsets.

### T cell activation in response to DC modulation

The presence of cytotoxic T cells in glioblastoma has been associated with improved patient outcome [59], thought to result from limiting tumor growth, but this depends on effective T cell activation. In our study, we therefore investigated the consequences of RCT-induced changes in cDC1- and cDC2-like cells on T cell activation and found a significantly higher T cell proliferation rate after chemoradiation of tumor cells, observed in co-cultures with both DC subsets. Despite this comparable qualitative effect, the proliferation rate of CD8^+^ T cells was higher than that of CD4^+^ T cells, consistent with greater activation of cDC1- than cDC2-like cells and their superior production of CXCL9/10 and other factors that support CD8+ T cell recruitment and function.

Conventional glioblastoma chemoradiation in our models not only exerts direct cytotoxic effects on tumor cells but also indirectly modulates antitumor immunity by influencing DC and T cell functions. After treatment, we observed a shift in the DC immune phenotype, with robust activation of cDC1-like cells, increased proliferation of CD8^+^ T cells, and higher cytokine and chemokine secretion compared to untreated conditions, supporting the idea that RT and TMZ cause DNA damage and tumor cell death, that releases tumor antigens and DAMPs, which are detected by DCs and translate into enhanced cross-priming and cytotoxic T cell responses.

Conversely, the relatively limited activation of cDC2-like cells in our system was associated with reduced CD4^+^ T cell proliferation, indicating that standard glioblastoma treatment promotes cDC1-like cell–CD8^+^ T cell pathways over cDC2-like cell–CD4^+^ T cell interactions. Overall, these findings support the idea that standard glioblastoma therapies not only reduce tumor size but also reshape the immune environment by altering dendritic cell activation and T cell responses in a subset-specific manner. However, the validity of these findings inside a physiological tumor remains to be established.

### Anti-PD-1 blockade and resistance

Several, not mutually exclusive, factors may explain the absence of a measurable effect of anti-PD-1 blockade in our assays. Clinical and preclinical studies have shown that immune checkpoint monotherapy has limited success in glioblastoma, indicating that PD-1/PD-L1 inhibition alone often cannot overcome the highly immunosuppressive environment of this disease. One possibility is that PD-1 blockade is most effective when a pool of functional, tumor-specific T cells already exists; however, in glioblastoma, both the frequency and functional status of such T cells may be suboptimal, even when their absolute numbers were sufficient in our *in vitro* system, given the experimental model. Additionally, although PD-L1 was detectable and further increased by irradiation in our model [[60], [61]], clinical data indicate that high PD-L1 expression is often required to achieve significant benefit from PD-1/PD-L1 inhibitors, suggesting that expression levels or spatial patterns in this patient-oriented setting may be insufficient to elicit a clear response. Importantly, PD-1/PD-L1 is just one of many active immunosuppressive pathways in glioblastoma, and other inhibitory receptors, suppressive cytokines and chemokines, metabolic constraints, and regulatory cell populations can sustain T cell dysfunction despite PD-1 blockade, contributing to both primary and acquired resistance [62]. Innate immune resistance refers to the activation of oncogenic pathways associated with a “cold” TME. In contrast, the induction of PD-L1 via pro-inflammatory cytokines, as seen with adaptive immune resistance, is linked to a “hot” TME. This kind of TME is associated with PD-1 pathway inhibition [63]. Investigating oncogenic signaling in a multi-factor in vivo setting could help address this issue. Also, compensatory immune checkpoint pathways or other resistance mechanisms, such as loss of antigenicity, may account for the lack of anti-PD-1 blockade effects. *In vivo*, increased regulatory T cell activity may also contribute to reduced efficacy of PD-1 blockade. Furthermore, standard chemoradiation can damage lymphocytes and reduce overall T-cell numbers, potentially further limiting the effectiveness of PD-1 blockade in enhancing effector responses. This effect may be magnified in a simplified *in vitro* co-culture system that does not fully replicate the intracranial TME, including the blood–brain barrier, stromal elements, and the complete range of immune cell subsets and checkpoints. These findings highlight the complexity of immune checkpoint regulation and indicate that the success of PD-1 blockade depends heavily on the context, requiring thorough evaluation of both tumor-intrinsic and extrinsic factors and motivating future studies into resistance mechanisms and rational combination of strategies to overcome them.

### Limitations and future directions

First, we want to emphasize that all experiments were conducted *in vitro* and ex vivo to gain, for the first time, mechanistic insights into how chemoradiation-treated glioblastoma cells affect subtypes of dendritic cells that are essential for triggering anti-tumor immune responses. However, complementary in vivo validation in tumor-bearing mouse models should be conducted in the future. Second, although our ex vivo models capture key aspects of DC–T cell crosstalk, they do not fully represent the complexity of the tumor environment, including stromal, vascular, and myeloid components that may influence RCT responses.

In our study, we utilized *in vitro*-differentiated cDC1- and cDC2-like cells as models to investigate DC function and T cell responses. While these cells are valuable tools for mechanistic studies due to their experimental accessibility and well-established protocols [[64], [65], [66]], they may not fully mirror the complexity and functional diversity of tissue-resident or tumor-infiltrating DCs found in vivo. These constraints limit our conclusions to mechanistic insights at the cellular level. Nonetheless, by systematically modeling multiple treatment and cell-intrinsic factors in a controlled setting, this study helps to disentangle individual mechanistic contributions to DC and T-cell modulation. Additionally, by defining how chemoradiation influences DC activation and subsequent T-cell responses, it provides a mechanistic rationale and experimental basis for future in vivo studies in tumor-bearing mice to test whether these effects translate into improved immune infiltration and therapeutic benefit.

## Conclusions

The treatment of glioblastoma remains a major challenge in radiation oncology. Over the past decades, many trails of new therapeutic interventions have been conducted to improve prognosis of patients with glioblastoma, but have not yet resulted in sufficient improvements. In particular, the experience with immunotherapeutic approaches in other tumor entities were promising but achieved only little in glioblastoma. Although glioblastoma is a highly immunosuppressive tumor, high rate of infiltrative immune cells suggests a therapeutic potential. Thus, it is necessary to further decipher cellular and molecular mechanisms underlying therapy success and resistance. Much experimental research has been conducted using different doses of radiation, often even without the addition of chemotherapeutic agents. However, little attention has been paid to the changes that these approaches induce in the tumor tissue beyond tumor cell death. Furthermore, the tumor material used to evaluate immune cell infiltration or molecule expression is often derived from untreated patients. In contrast, our data suggest different responses of immune cell activation and subsequent T cell proliferation depending on the particular treatment regimen and DC subtype. In particular, chemoradiation as a combination therapy had the most pronounced effect on cDC1-like cells and was beneficial in promoting a tumor cell-induced T cell proliferation in a proinflammatory manner. Therefore, further (preclinical) studies should be designed by implementing current therapeutic strategies and thus mimic the pathological situation as closely as possible. This will give the chance to integrate distinct immune therapies and modulations in the multimodal therapy of glioblastoma.

## Funding statement

This work has been funded by the Deutsche Forschungsgemeinschaft (DFG, German Research Foundation, project number DE 2503/2-1).

## CRediT authorship contribution statement

**Celina Schuster:** Formal analysis, Investigation, Methodology, Validation, Visualization, Writing – original draft, Writing – review & editing. **Benjamin Frey:** Formal analysis, Resources, Validation, Writing – review & editing. **Rainer Fietkau:** Resources, Writing – review & editing. **Stefanie Corradini:** . **Udo S. Gaipl:** Conceptualization, Formal analysis, Project administration, Resources, Supervision, Validation, Writing – original draft, Writing – review & editing. **Anja Derer:** Conceptualization, Data curation, Formal analysis, Funding acquisition, Investigation, Methodology, Project administration, Supervision, Validation, Visualization, Writing – original draft, Writing – review & editing.

## Declaration of competing interest

The authors declare that they have no known competing financial interests or personal relationships that could have appeared to influence the work reported in this paper.
