# Supplementary material for: Chemoradiation of glioblastoma cells alters expression of activation and immune checkpoint molecules on type 1 and 2 dendritic cells and impacts on subsequent T cell proliferation
Source: Clin Transl Radiat Oncol. 2026 Jan 9;57:101102. doi: 10.1016/j.ctro.2025.101102 (PMC12861272; doi:10.1016/j.ctro.2025.101102)
Supplement: Supplementary Data 1 [file mmc1.pdf]

A

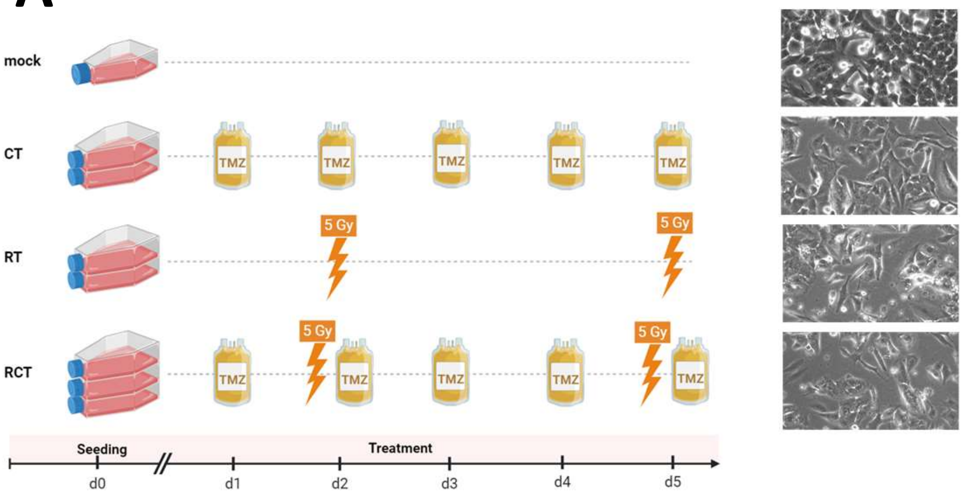

B

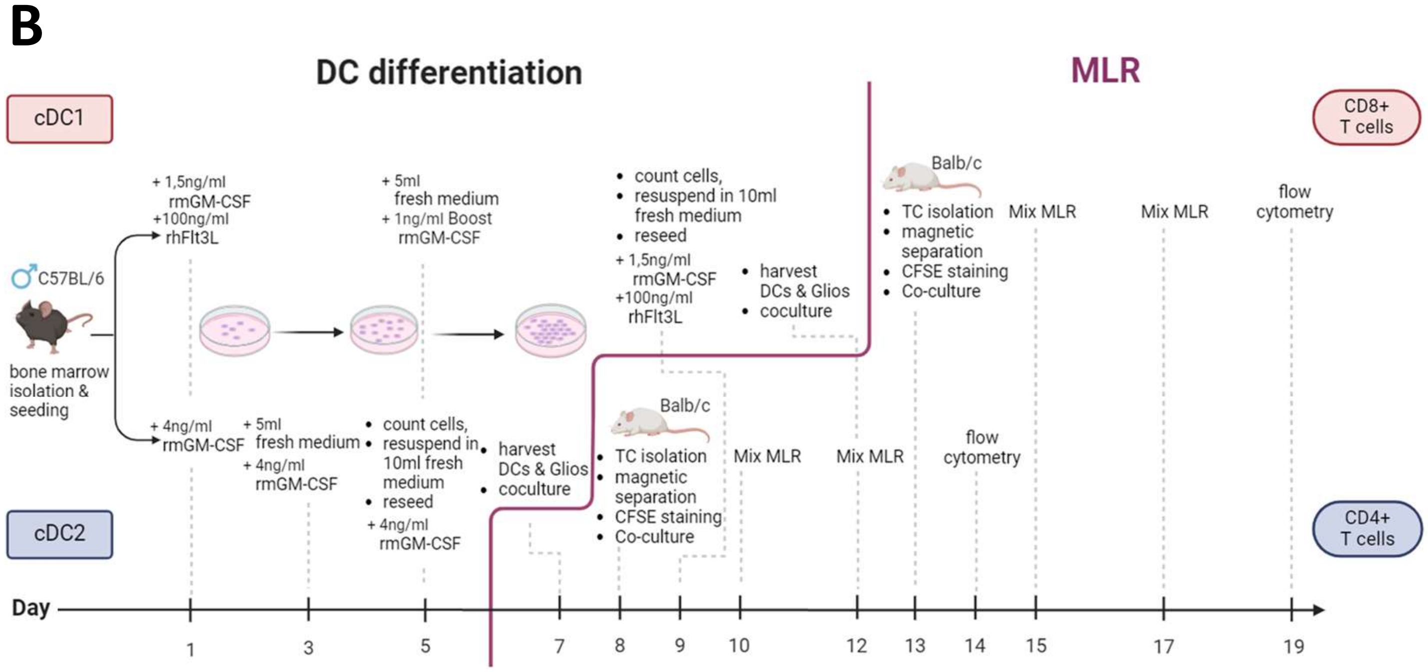

Supplementary Figure S1: Workflow of the treatment scheme of (A) GL261-luc2 glioblastoma cells and (B) cDC1 and cDC2 differentiation and mixed lymphocyte reaction
