# Supplementary figures and images for: Chemoradiation of glioblastoma cells alters expression of activation and immune checkpoint molecules on type 1 and 2 dendritic cells and impacts on subsequent T cell proliferation
Source: Clin Transl Radiat Oncol. 2026 Jan 9;57:101102. doi: 10.1016/j.ctro.2025.101102 (PMC12861272; doi:10.1016/j.ctro.2025.101102)

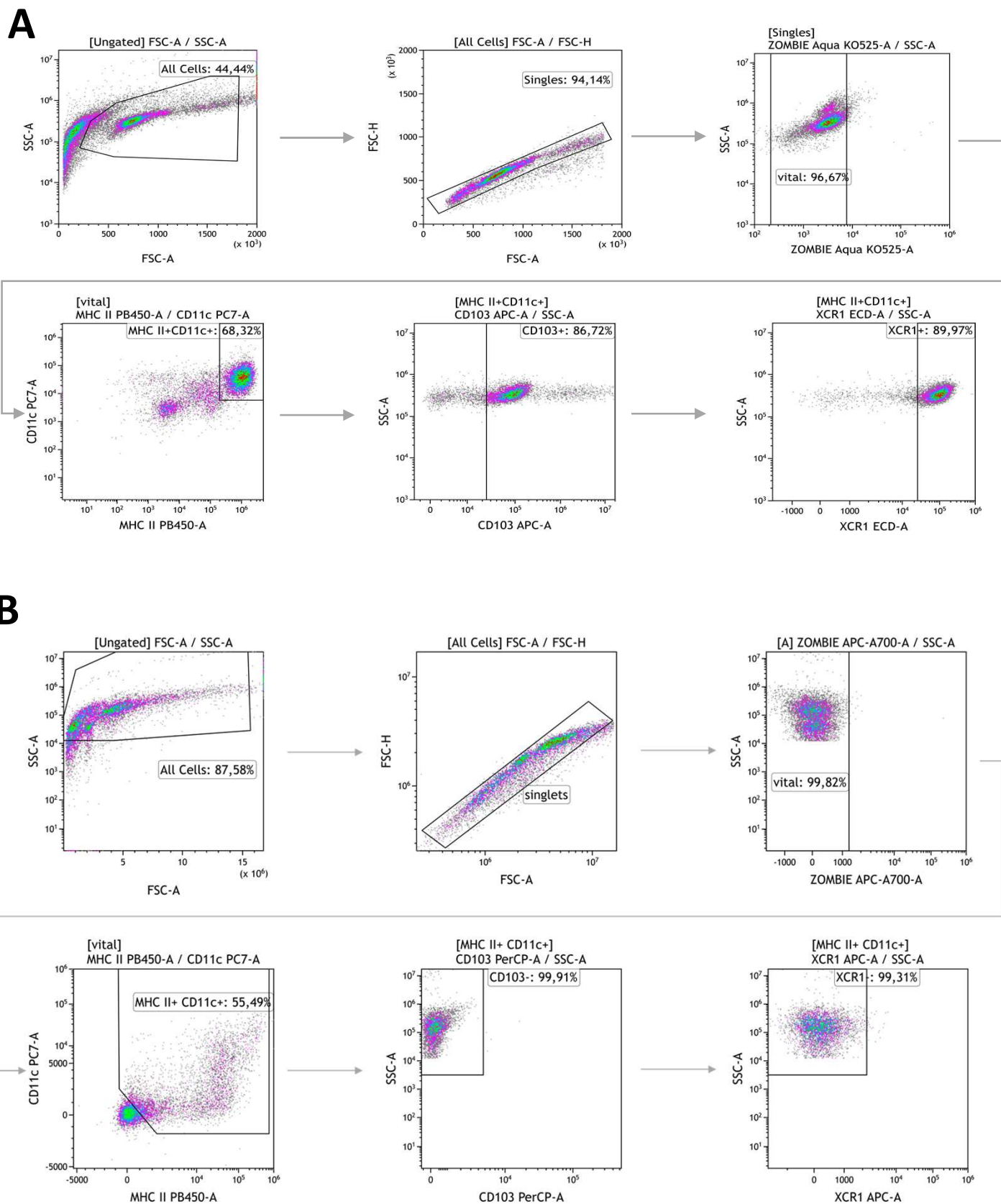

**Supplementary Figure S2: Flow cytometric gating strategy for cDC differentiation.**

Supplement: Supplementary Data 2 [file mmc2.pdf]

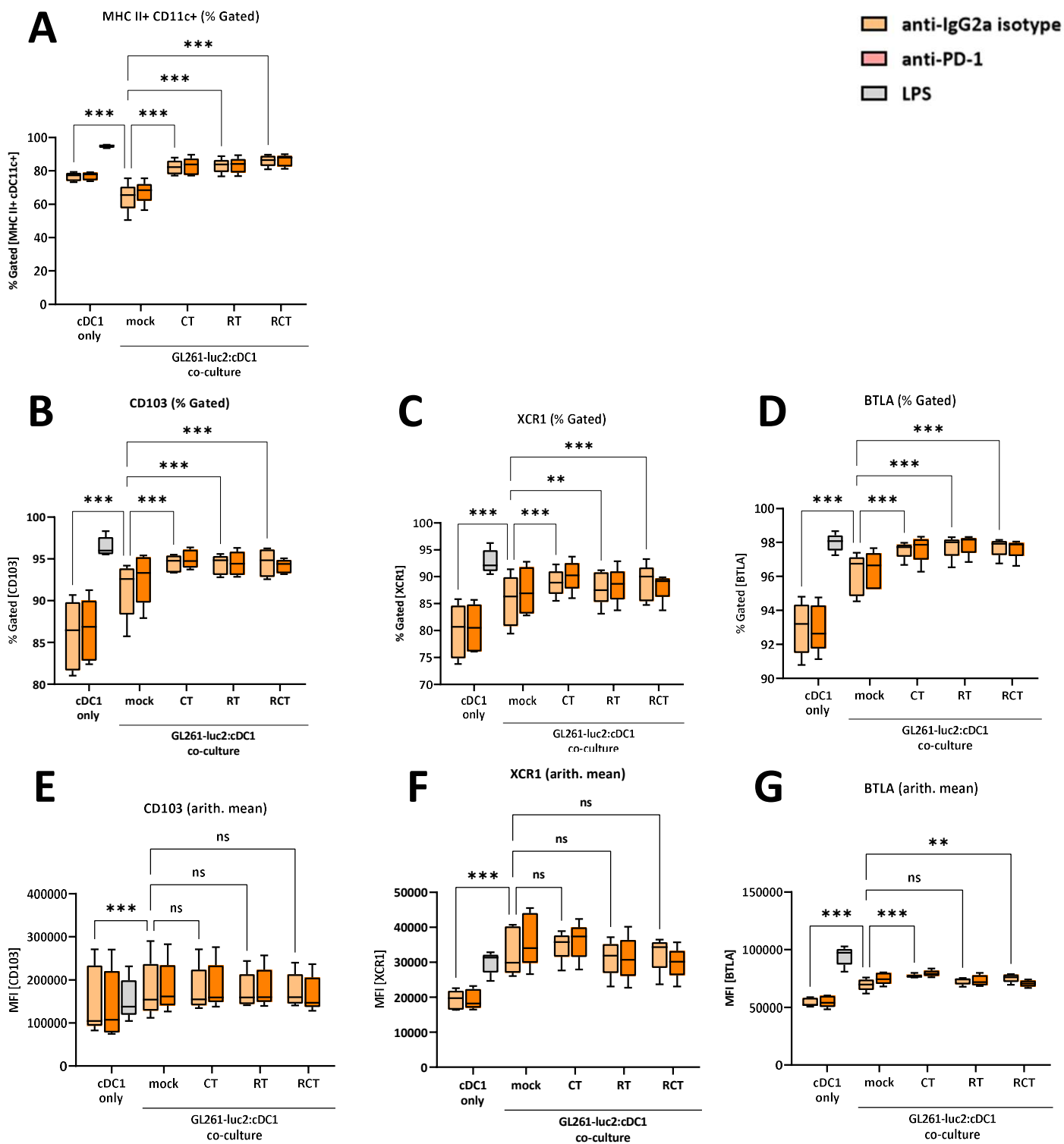

Supplement: Supplementary Data 3 [file mmc3.pdf]
