# Supplementary material for: Chemoradiation of glioblastoma cells alters expression of activation and immune checkpoint molecules on type 1 and 2 dendritic cells and impacts on subsequent T cell proliferation
Source: Clin Transl Radiat Oncol. 2026 Jan 9;57:101102. doi: 10.1016/j.ctro.2025.101102 (PMC12861272; doi:10.1016/j.ctro.2025.101102)
Supplement: Supplementary Data 4 [file mmc4.pdf]

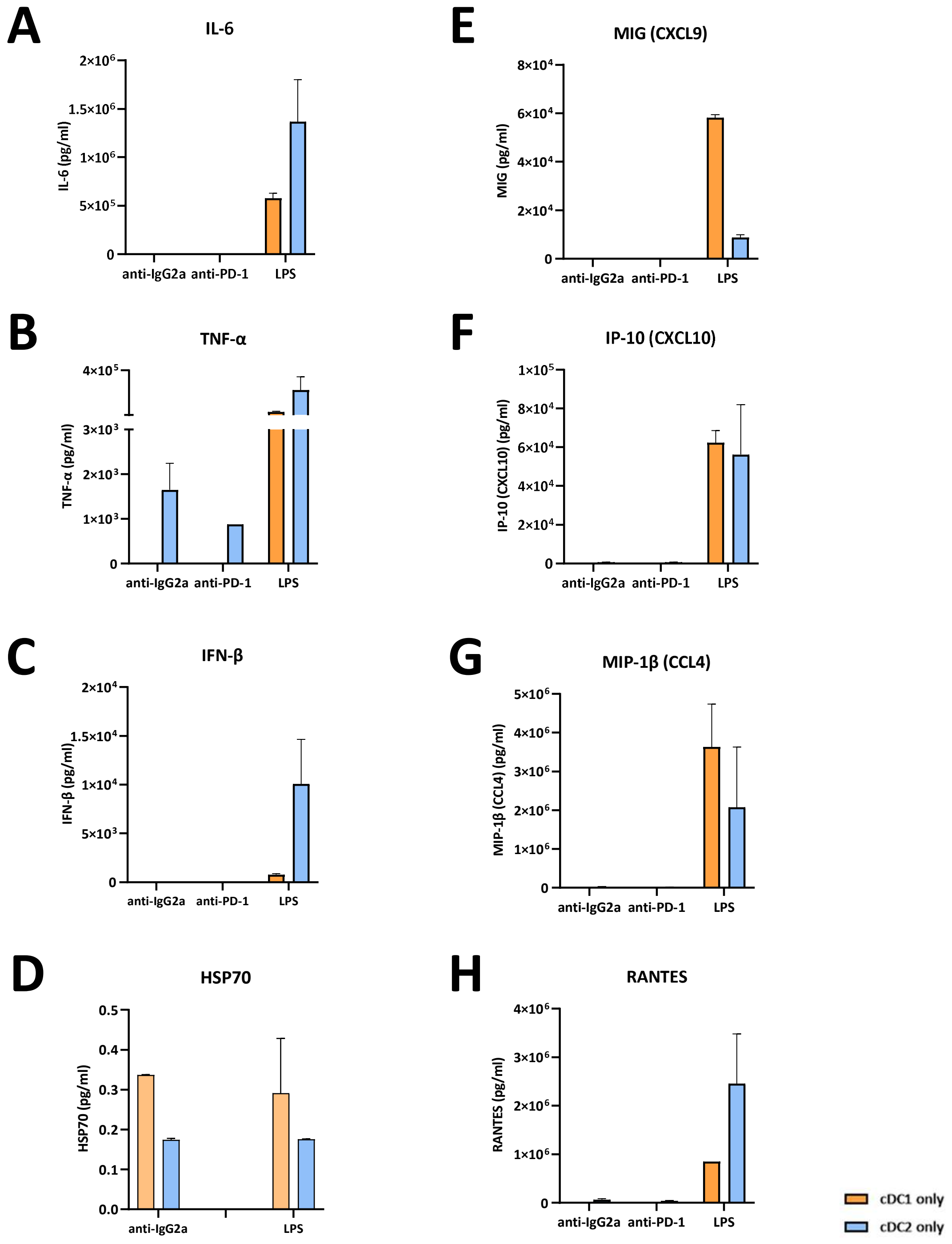

Supplementary Figure S4: Analyses of stimulatory capacity of mono-culture of predifferentiated cDC1- and cDC2 like cells after incubation with isotype antibody (anti-IgG2a), PD-1 blockade (anti-PD-1) and LPS. Secretion level (pg/mL) for (A) IL-6, (B) TNF-α, (C) IFN-β, (D) HSP70, (E) MIG, (F) IP-10, (G) MIP-1β, and (H) RANTES.
