# Supplementary material for: Chemoradiation of glioblastoma cells alters expression of activation and immune checkpoint molecules on type 1 and 2 dendritic cells and impacts on subsequent T cell proliferation
Source: Clin Transl Radiat Oncol. 2026 Jan 9;57:101102. doi: 10.1016/j.ctro.2025.101102 (PMC12861272; doi:10.1016/j.ctro.2025.101102)
Supplement: Supplementary Data 5 [file mmc5.pdf]

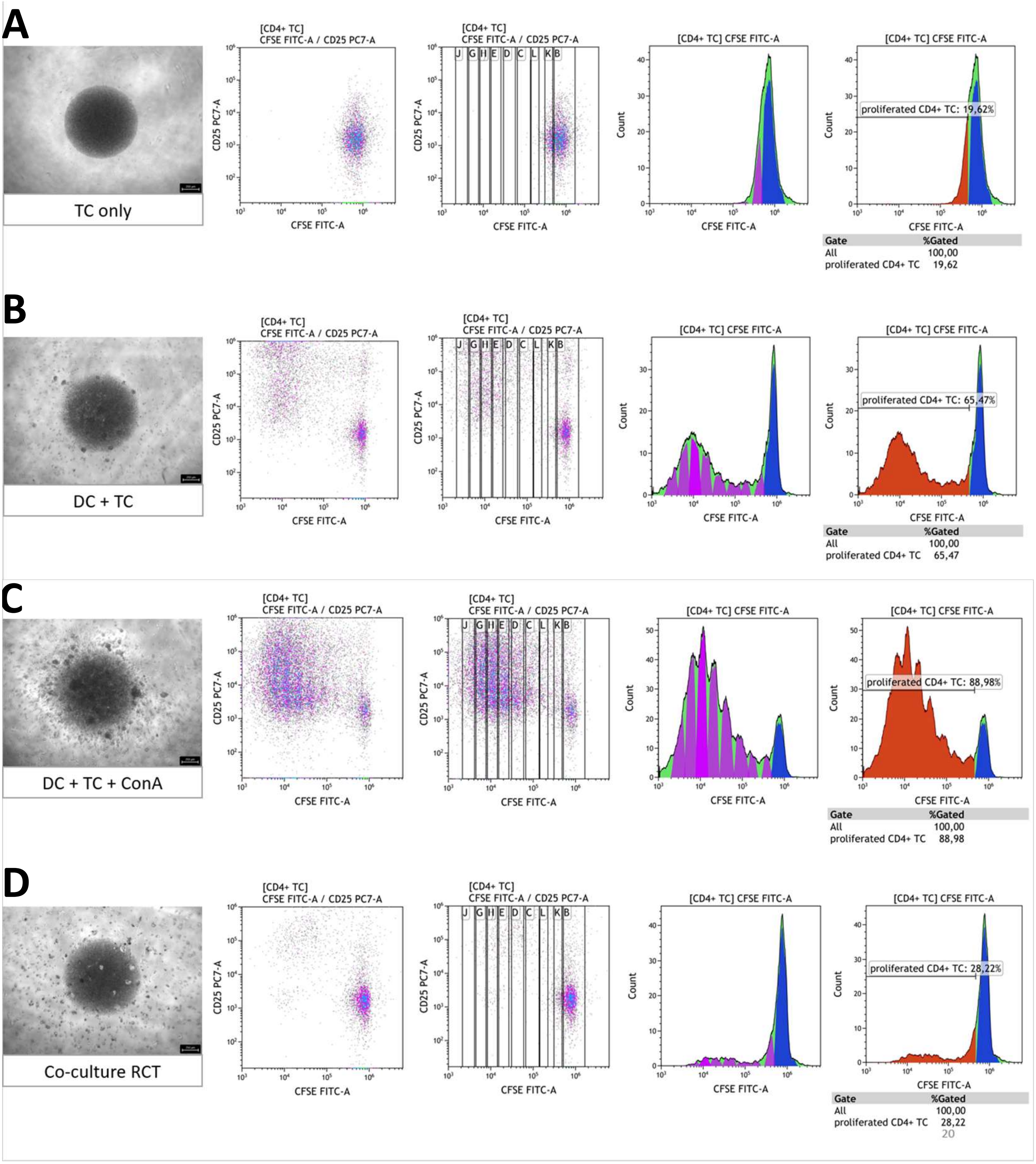

Supplementary Figure S5: Light microscopy photographs and gating strategy of the mixed lymphocyte reaction.
